# Pitch then power: limitations to acceleration in quadrupeds

**DOI:** 10.1098/rsbl.2009.0360

**Published:** 2009-06-24

**Authors:** Sarah B. Williams, Huiling Tan, James R. Usherwood, Alan M. Wilson

**Affiliations:** Structure and Motion Laboratory, The Royal Veterinary College, University of London, Hatfield, Herts AL9 7TA, UK

**Keywords:** acceleration, muscle power, pitch, biomechanics, morphology

## Abstract

Rapid acceleration and deceleration are vital for survival in many predator and prey animals and are important attributes of animal and human athletes. Adaptations for acceleration and deceleration are therefore likely to experience strong selective pressures—both natural and artificial. Here, we explore the mechanical and physiological constraints to acceleration. We examined two elite athletes bred and trained for acceleration performance (polo ponies and racing greyhounds), when performing maximal acceleration (and deceleration for ponies) in a competitive setting. We show that maximum acceleration and deceleration ability may be accounted for by two simple limits, one mechanical and one physiological. At low speed, acceleration and deceleration may be limited by the geometric constraints of avoiding net nose-up or tail-up pitching, respectively. At higher speeds, muscle power appears to limit acceleration.

## 1. Introduction

Acceleration requires power from muscles to increase the kinetic energy of the centre of mass (CoM). The muscle-specific powers associated with near-maximal accelerations for a range of bipeds are high: accelerating turkeys (Roberts & Scales 2002) can reach a mean of 55–60 W kg^−1^ over a complete gait cycle. Similar values are achieved by sprinting humans (Cavagna *et al*. 1971; Janssen *et al*. 2000). Accelerating wallabies (McGowan *et al*. 2005) achieve 114 W kg^−1^ hindlimb muscle mass-specific power for a complete gait cycle (although this is probably an overestimate as it ignores power from trunk muscles). Therefore, muscles have been reasonably hypothesized as performing maximally during maximal accelerations (Roberts & Scales 2002). Our interest here is whether muscle power presents the single limit accounting for maximal acceleration and deceleration. If so, the main selective pressure concerning acceleration is the amount of power locomotor muscles can produce and the reduction of non-muscle mass.

**Figure 1.**
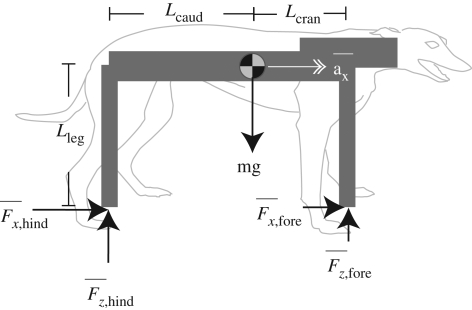
Free-body diagram of the stride-average forces acting on a generic quadruped of unvarying body geometry, assuming acceleration/deceleration are powered predominantly by limb torques. ⊕ denotes CoM, 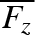 vertical ground reaction force (opposes weight), and 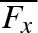 horizontal force that accelerates/ decelerates (−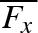) the animal. We consider only 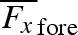 during braking and 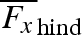 during propulsion. *L*_leg_ is the length of leg, *L*_cran_ and *L*_caud_ are the distances from CoM to hip/shoulder joint, respectively.

**Figure 2.**
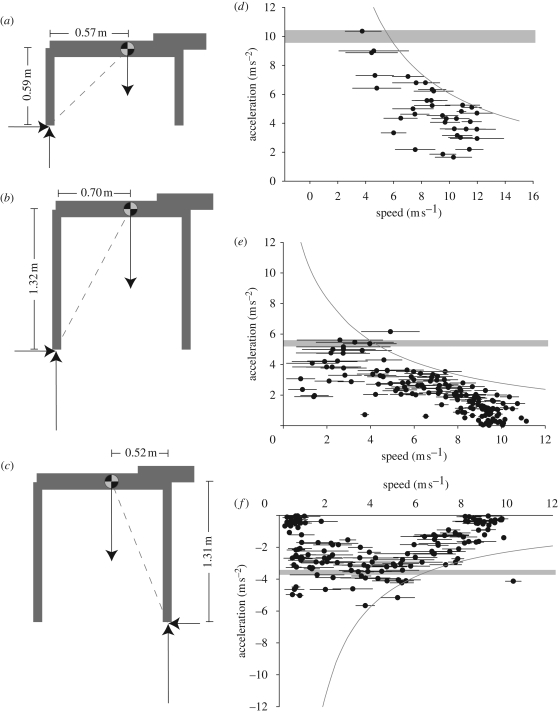
(*a*–*c*). The body geometry used to create limiting stride-averaged accelerations for maximally accelerating greyhounds and accelerating and decelerating horses. The maximum net nose-up (*a*,*b*) or tail-up (*c*) pitching acceleration is determined by constraining the total ground force vector (dotted line) to be at the mean (mid-stance) hind (*a*,*b*) or fore (*c*) foot position and assuming that geometry is largely constant. The resulting maximum acceleration (*d*,*e*) and deceleration (*f*) predictions are indicated by horizontal grey bars. Bar thickness denotes 1 s.d. due to the range of body geometry measured (*n* = 5, horses and dogs). (*d*) Acceleration data for greyhounds (70 strides, 10 dogs). (*e*) Accelerating polo ponies (160 strides, five ponies). (*f*) Decelerating polo ponies (160 strides, five ponies). Each data point indicates mean speed and mean acceleration for a single stride; bar ends denote initial and final velocity of the stride. At low speeds, maximal accelerations are consistent with pitch avoidance. At higher speeds, lower maximum accelerations are achieved, consistent with a power constraint. Body-mass specific power requirements for the curves are 60, 30 and −23 W kg^−1^ for figure parts *d*–*f*, respectively.

Accelerating quadrupeds may, by analogy with accelerating motorcycles, be limited by two further, inter-related physical constraints: pitch avoidance and traction. Just as an increase in accelerating torque in a motorcycle results in nose-up pitching and reduced weight support on the front wheel, a similar principle applies to accelerating quadrupeds (where the hind leg is equivalent to a spoke of the rear wheel and most of the propulsive musculature is arranged to retract, or pull the leg backwards). In figures 1 and 2*a*–*c*, we present a free-body diagram of hypothesized forces during maximal accelerations of quadrupeds. The model (see electronic supplementary material, pitch-avoidance model) is similar to that proposed by Gray (1944) for animals standing on inclined surfaces and is similarly simplified by applying the following assumptions and constraints: (i) a net pitching acceleration over a stride is avoided; (ii) the body geometry is constant: this assumption is deliberately simplistic, ignoring motions of the head (and tail) with respect to the CoM (contrasting with a similar formulation expressed for lizards (Aerts *et al*. 2003)); and (iii) accelerations are presumed to be powered by torque of the limb (about shoulder and/or hips), rather than limb extension (Biewener 1989; Williams *et al*. 2009)—hence the feet are drawn directly beneath the hip/shoulder in figures 1 and 2. This results in a constraint to horizontal acceleration, 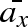, as the net force vector over a stride, during maximal acceleration (propulsion) must pass through or behind the CoM (see figure 1 and the electronic supplementary material):
1.1
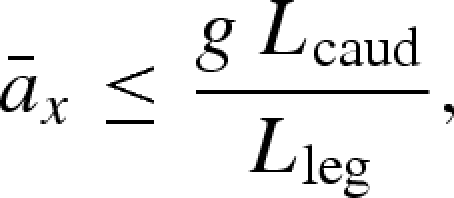

where *g* is the acceleration caused by gravity, *L*_caud_ the distance between hips and CoM and *L*_leg_ the length of the hind leg.

Here, we use this model in conjunction with experimental measurements of racing greyhound and polo pony (horse) accelerations in order to determine whether, and when, power or pitch-avoidance might constrain acceleration in quadrupeds.

## 2. Material and methods

### (a) Morphological measurements

All measurements for the pitch-avoidance model (figures 1 and 2*a*–*c*) were taken during quiet standing of five greyhounds and five horses. *L*_leg_ denotes distance from foot to greater trochanter of femur (hip) (when considering accelerations) and foot to greater tubercle of humerus (shoulder) (for decelerations). *L*_caud_ denotes the distance from hip to CoM, derived from the distance measured between hip and cranial aspect of the shoulder, and published front-hind weight bias during steady locomotion (0.56 for dogs (Lee *et al*. 1999) and 0.57 for horses (Witte *et al*. 2004)).

### (b) Greyhounds

Ten race-fit greyhounds, each performing a single acceleration from racing traps, were filmed (250 fps, 1280 × 256 resolution, Troubleshooter HR, Fastec imaging, San Diego, USA) through a calibrated space (average of 70 pixels m^−1^) during pre-race trials. Digitized motions of a CoM proxy were smoothed (least-squares spline filter; Walker 1998) and differentiated to provide velocity and acceleration; means over a stride are reported. Mean body and muscle mass-specific power requirements were also determined from kinematics (maximum constraining power taken as the maximum of the products of mean speed and mean acceleration over each stride) and the known body/muscle masses of racing greyhounds (Williams *et al*. 2008).

### (c) Polo ponies

Five competition polo ponies were ridden by a professional rider to perform five maximal accelerations and five maximal decelerations in an all-weather polo arena. Body velocities and accelerations were derived from GPS and inertial sensor measurements: velocities over each of 160 strides (30–40 per horse) in acceleration and 160 strides in deceleration were measured using high performance dual frequency carrier wave differential GPS (20 Hz update rate, 0.02 m horizontal position accuracy, 0.03 m s^−1^ speed accuracy, OEM4 Novatel, Calgary, Canada, postprocessed in Grafnav 7.60, Waypoint, Calgary, Canada). High-frequency (within stride) changes in velocity and position of the horse relative to the rider were tracked by integrating measurements from an inertial measurement unit (MTx Xsens Enschede, The Netherlands (Pfau *et al*. 2005)), mounted on the trunk of the horse. Both types of sensor data were combined to give increased measurement accuracy (Tan *et al*. 2008). Stride timings were derived from hoof-mounted 50 g accelerometers logged into MP3 recorders (Parsons & Wilson 2006).

## 3. Results and discussion

### (a) Morphological measurements

Greyhound *L*_leg_ = 0.59 ± 0.04 m (mean±s.d. throughout), and *L*_caud_ = 0.57 ± 0.03 m. Polo pony, forelimb *L*_leg_ = 1.31 ± 0.02 m, and hindlimb *L*_leg_ = 1.32 ± 0.03 m. *L*_caud_ and *L*_cran_ were 0.70 ± 0.02, and 0.52 ± 0.02 m, respectively.

### (b) Acceleration

In both species maximum acceleration dropped with speed (figure 2*d*–*f*). The theoretical pitch-avoidance limit is shown as horizontal bars (thickness denotes 1 s.d.); constant power requirement limits as curves. At speeds above 5 m s^−1^, maximum accelerations are consistent with constant, constrained power availability. The muscle powers required to drive maximal accelerations are very high: the limiting lines represent 60 W kg^−1^ (greyhounds), and 30 W kg^−1^ (ponies) in body mass specific terms—very high compared with the 8–13 W kg^−1^ measured for bipeds (Cavagna *et al*. 1971; Roberts & Scales 2002), and similar to the CoM powers of ascending quail (Askew *et al*. 2001; 65 W kg^−1^). Using published values for muscle mass as a proportion of body mass (greyhounds 50% (Williams *et al*. 2008), horse 40% (hence horse + rider 35%, assuming no rider contribution to power)), muscle mass-specific power is calculated as 120 and 85 W kg^−1^ for greyhound and pony, respectively. These values are very high for cyclic activity, supporting the suggestion that muscles may be operating at maximal power during maximal acceleration at high speeds. Mass specific muscle power should be directly measured in order to establish unequivocally whether this is the case.

At low speeds, the power requirement curve fails to account for maximal accelerations. Instead, the data fall within or below the linear limit predicted by the pitch-avoidance model. At low speeds, this limit is reached but, crucially, not exceeded by both greyhounds and horses: maximal accelerations were 10 and 6 m s^−2^, respectively. Video illustrates that such accelerations can cause net nose-up pitch (see movie S1, electronic supplementary material): the forelimbs barely contact the ground during the stride and the trunk pitches up during hindlimb contact. This may initially appear to contradict the suggestion that accelerations cannot breach the theoretical ‘pitch-limit’, however, the animal is unrestricted by the various constraints of the model.

### (c) Deceleration

Results for deceleration in ponies largely mirror those of acceleration. There appears to be a reduced capacity for deceleration at higher speeds, suggesting a ‘negative’ or ‘dissipative’ power constraint of approximately −23 W kg^−1^ of horse + rider mass (−27 W kg^−1^ of horse mass). With little *a priori* knowledge of the functional muscle mass for deceleration, however, this is difficult to interpret further. An alternative explanation may be that at high speeds, horses may begin deceleration with a ‘preparatory’ stride in order to get ready for substantial braking.

At lower speeds, decelerations occasionally exceeded those predicted by the pitch-avoidance model; in this case, the assumption of unvarying geometry is clearly broken. Indeed, during rapid deceleration, both the rider and the pony's head and neck move backwards. Such motions can, however, be understood within the context of the simple pitch-avoidance model since this moves the CoM towards the hips, increasing *L*_cran_.

### (d) Context

The pitch-avoidance and power-limit models, while simple, provide a framework for understanding behavioural and anatomical adaptations for extreme accelerations, sometimes by simply highlighting when the three key assumptions are being broken. (i) *Muscle power*. Very high accelerations are possible in jumping (locusts (Bennet-Clark 1975), galagos (Aerts 1998) and froghoppers (Burrows 2006)), as mechanical power is not limited by muscle power because energy is stored in elastic elements, and released quickly during the leap. (ii) *Pitch-avoidance*. Springtails achieve high acceleration jumps, but break the assumption of zero net change in angular velocity: they spin rapidly backwards during their ballistic flight (Brackenbury & Hunt 1993). (iii) *Constant geometry*. Most jumpers and accelerating bipeds (Roberts & Scales 2002) avoid pitching during high accelerations by aligning the resultant force vector through the CoM and powering through limb extension using several joints.

Traction may, on occasion, also present a constraint to acceleration; however, slipping was not observed, and greater centripetal accelerations are achieved for both greyhounds and ponies when running around bends (14 m s^−2^ (from Usherwood & Wilson 2005) and 8 m s^−2^, respectively). Alternative factors, particularly muscle mechanics, cannot be discounted from providing constraints to acceleration capacity at low speeds. Assuming the effective mechanical advantage of the limb is constrained to some degree, then high forces applied during accelerations at low-intermediate speeds might restrict the availability of muscle power. This is because force-velocity properties of muscles predict higher muscle forces at low contraction velocities and maximum muscle powers to occur at about 31 per cent of maximum shortening speed (Hill 1938). Additionally, the assumptions imposed for our model can be seen, to a certain extent, to be broken (hind legs do not produce purely torquing forces, nor is the CoM rigidly connected to the hips). However the pitch-constraint model has the benefit of providing a reductionist, parsimonious and, remarkably, predictive account for the observation of reduced acceleration capacity at low speeds.
